# Supplementary material for: Identification and Characterization of Circular RNAs in Brassica rapa in Response to Plasmodiophora brassicae
Source: Int J Mol Sci. 2022 May 11;23(10):5369. doi: 10.3390/ijms23105369 (PMC9141718; doi:10.3390/ijms23105369)
Supplement: Supplementary file 1 [file ijms-23-05369-s001.zip › Supplementary document.pdf]

Supplementary document

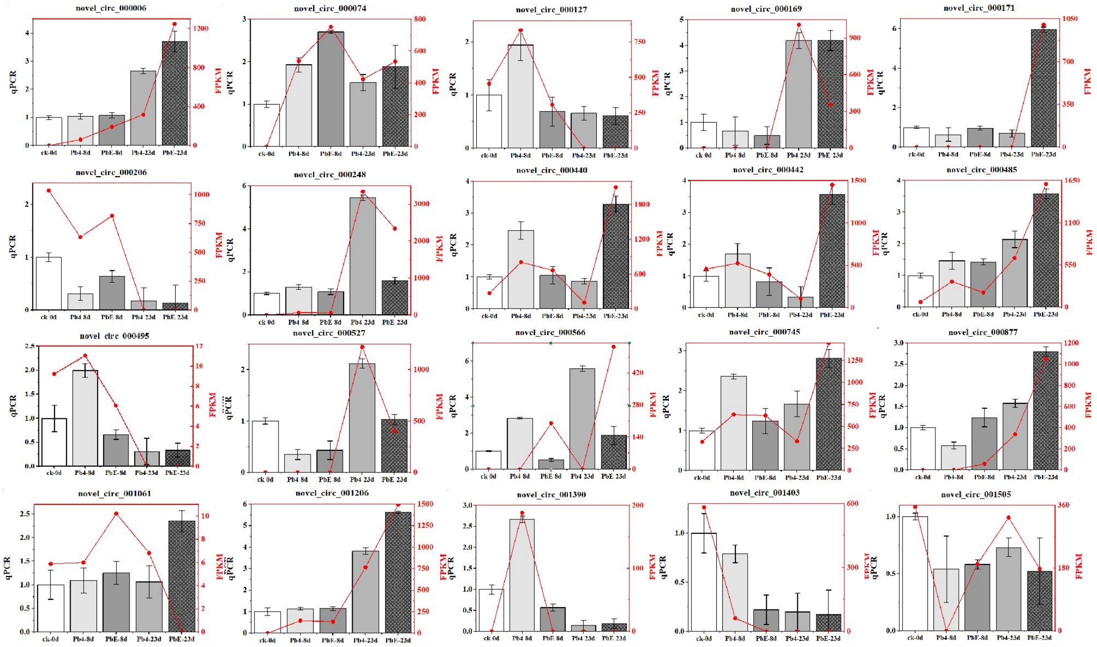

Figure S1. Quantitative real-time PCR validation for RNA-seq.

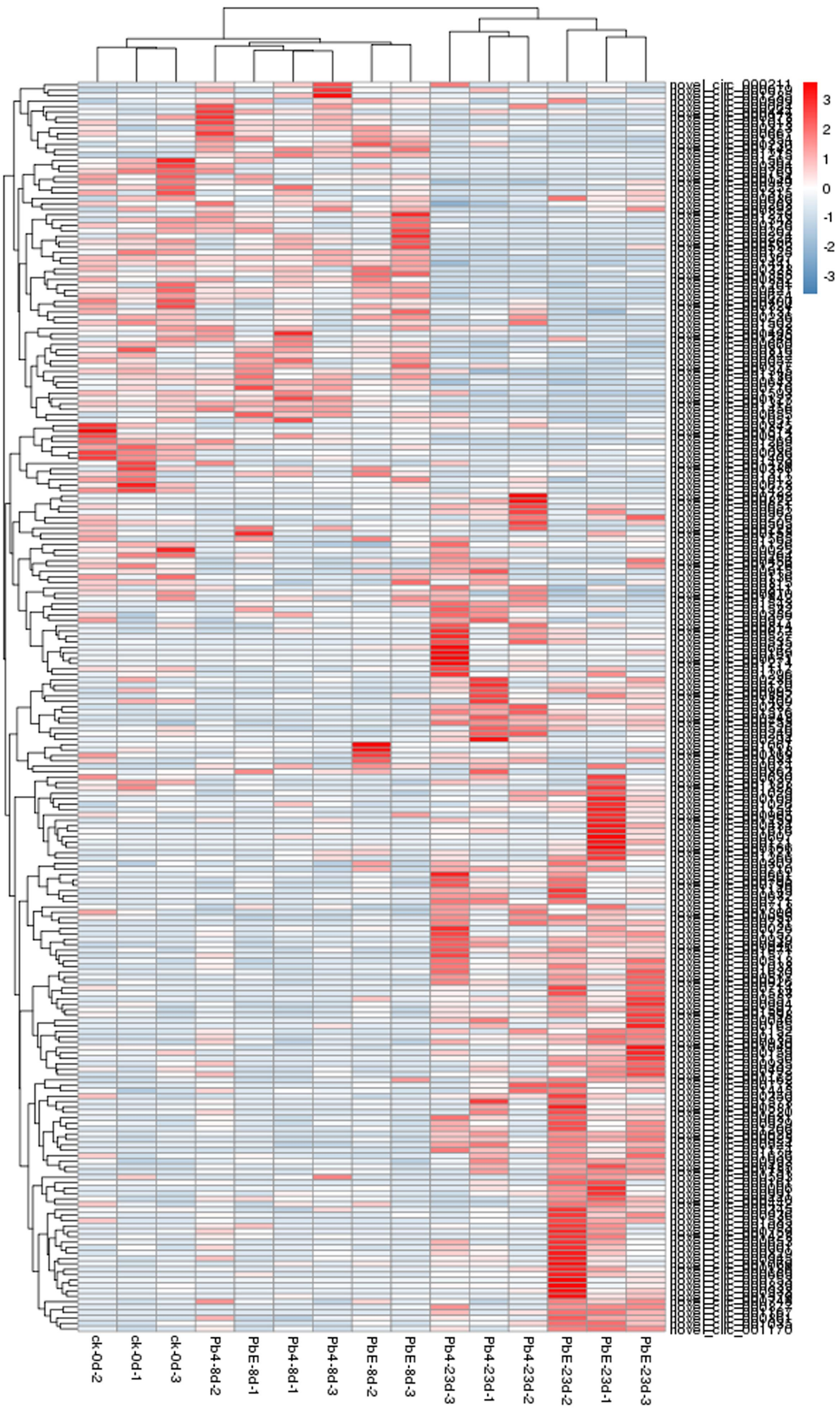

Figure S2. Heat map and clustering of circRNA expression profiles in different samples.

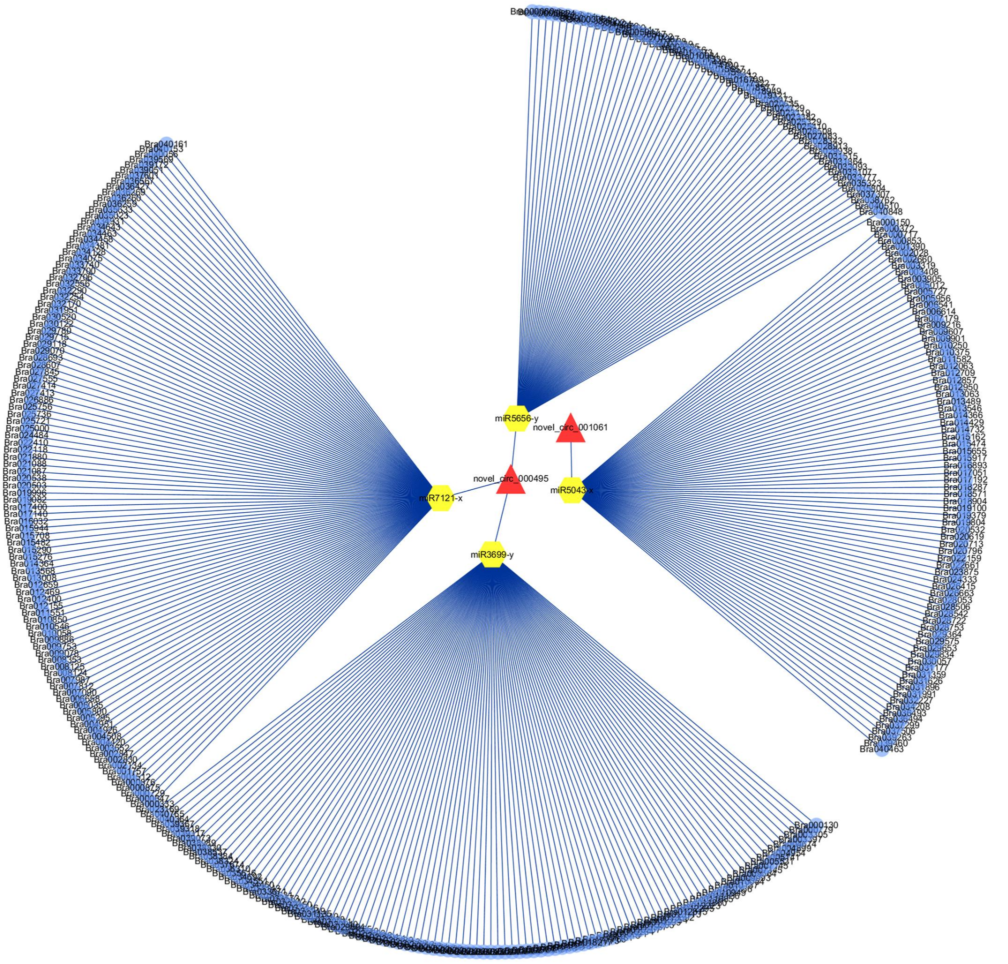

Figure S3. View of the circRNA – miRNA – mRNA triple network Brassica rapa. The circRNAs, miRNAs, and mRNAs are indicated as yellow hexagons, red triangles, and blue diamonds, respectively.

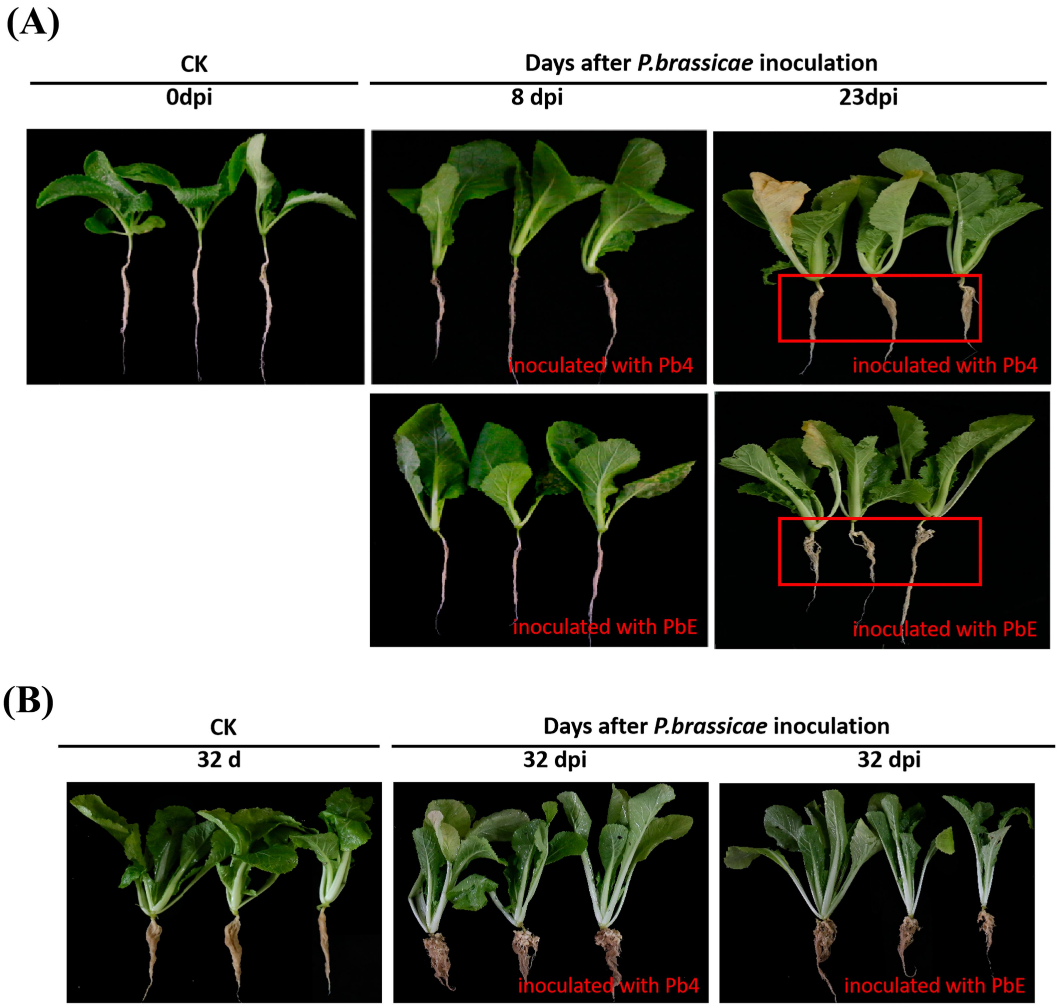

Figure S4. (A) Phenotype of roots in 'BJN 222' plants infected with *P. brassicae*. (B) Phenotype of roots in 'BJN 3-2' plants infected with *P. brassicae*.
